# Circulating tumor DNA in Hodgkin lymphoma: biological classification, molecular residual disease, and integration with PET response

**DOI:** 10.3389/fonc.2026.1982526

**Published:** 2026-10-29

**Authors:** Marta Bednarek, Maria Komossa, Joanna Bidzińska

**Affiliations:** 1 2nd Department of Radiology, Medical University of Gdańsk, Gdańsk, Poland; 2 Central Clinical Laboratory, University Clinical Centre in Gdańsk, Gdańsk, Poland; 3 Faculty of Health Sciences, Medical University of Gdańsk, Gdańsk, Poland; 4 Radiology Department, University Clinical Centre in Gdańsk, Gdańsk, Poland

**Keywords:** cell-free DNA, circulating tumor DNA, FDG-PET, hematologic malignancies, Hodgkin lymphoma, liquid biopsy, minimal residual disease, molecular response

## Abstract

Circulating cell-free DNA (cfDNA) and its tumor-derived fraction, circulating tumor DNA (ctDNA), enable non-invasive tumor genotyping, longitudinal disease monitoring, and molecular residual disease (MRD) assessment. Classical Hodgkin lymphoma (cHL) is particularly suitable for ctDNA-based analysis because malignant Hodgkin and Reed-Sternberg cells usually constitute only a small fraction of the tumor mass, whereas plasma may contain a strong tumor-derived DNA signal. This narrative review summarizes the potential applications of cfDNA-based ctDNA analysis in hematologic malignancies, with a focused emphasis on cHL. We discuss pre-analytical and analytical requirements, including plasma processing, tumor-informed and tumor-naive sequencing, error suppression, droplet digital PCR, and control for clonal hematopoiesis. We then synthesize evidence for ctDNA as a dual-readout biomarker: a baseline tool for non-invasive biological classification and a dynamic measure of molecular response. Published studies identify cHL genomic subtypes, associations with the tumor microenvironment, and prognostic information complementary to PET and conventional risk factors. Age-specific evidence is uneven, and most studies demonstrate analytical feasibility or prognosis rather than benefit from a ctDNA-triggered intervention. ctDNA-MRD should complement FDG-PET/CT; it cannot localize disease or currently replace established staging and response criteria. Routine implementation will require harmonized sample handling, validated assays and reporting thresholds, external quality assessment, and prospective trials demonstrating that ctDNA-guided treatment improves disease control, toxicity, or quality of life.

## 1 Introduction

Precision oncology increasingly depends on the ability to define tumor biology, measure treatment response, and detect residual disease before clinical relapse becomes apparent. In hematologic malignancies, these goals have traditionally been pursued through tissue biopsy, bone marrow assessment, imaging, flow cytometry, cytogenetics, and targeted molecular assays. Tissue biopsy remains indispensable for diagnosis and histologic classification, whereas FDG-PET/CT is central to staging and response assessment in cHL. Biopsy is invasive and may underrepresent spatial and temporal tumor heterogeneity when only a single lesion is sampled (1–7).

Liquid biopsy offers a complementary strategy by analyzing tumor-derived components in peripheral blood or other body fluids. Cell-free DNA (cfDNA) denotes all extracellular DNA fragments in plasma, derived predominantly from hematopoietic cells but also from other normal tissues and, in patients with cancer, malignant cells. Circulating tumor DNA (ctDNA) is specifically the tumor-derived fraction of cfDNA; the terms are therefore related but not interchangeable. Apoptosis and necrosis are major sources, with active release and extracellular DNA processing also contributing. ctDNA contains tumor-associated somatic single-nucleotide variants, insertions/deletions, copy-number alterations and structural variants, and can carry informative methylation and fragmentation patterns. In cHL, tumor-specific alterations originate from HRS cells, whereas DNA released by nonmalignant microenvironmental cells contributes to the background cfDNA pool (4–9).

The quantity of ctDNA varies with tumor burden, cell turnover, anatomical distribution, treatment, and assay sensitivity. A high total cfDNA concentration does not necessarily imply a high ctDNA concentration. Results may be reported as variant allele fraction (VAF), tumor fraction, mutant molecules per milliliter, or haploid genome equivalents per milliliter (hGE/mL); these measurements are not directly interchangeable. For illustration, baseline ctDNA estimates in the prospective Camus cohort ranged from 0 to 2684.9 hGE/mL (10). Serial tumor-specific measurements can support non-invasive genotyping, assessment of molecular burden, response monitoring, detection of emerging resistance, and investigation of relapse, while a negative result remains dependent on sample input and assay sensitivity (5, 8).

The clinical value of ctDNA is well established in several solid tumors and is rapidly expanding in hematologic malignancies. In lymphomas, ctDNA can support molecular subtyping, estimate disease burden, monitor early molecular response, identify resistant clones, and detect relapse. In cHL, ctDNA is especially attractive because conventional tissue-based genomic profiling is limited by the scarcity of malignant Hodgkin and Reed-Sternberg (HRS) cells within a complex inflammatory microenvironment. This biological paradox makes cHL one of the most compelling settings for liquid-biopsy-based molecular profiling (5–8).

cHL is a mature B-cell neoplasm in which HRS cells generally originate from germinal-center B cells but lose much of the normal B-cell transcriptional program. Recurrent abnormalities activate survival pathways, including NF-kB and JAK/STAT, and impair antigen presentation. Alterations of 9p24.1, containing PD-L1, PD-L2, and JAK2, and Epstein-Barr virus (EBV) infection in a subset of tumors contribute to immune escape. HRS-derived cytokines and chemokines recruit supportive immune and stromal cells, creating a microenvironment that is central to disease biology (1, 2, 9, 11).

First-line treatment depends on stage, risk, fitness, and access to therapy. In younger fit adults, ABVD (doxorubicin, bleomycin, vinblastine, and dacarbazine), often followed by PET-adapted omission of bleomycin, remains an established approach. For advanced-stage disease, brentuximab vedotin plus AVD (BV-AVD) and PET-guided BrECADD (brentuximab vedotin, etoposide, cyclophosphamide, doxorubicin, dacarbazine, and dexamethasone) are evidence-based options; HD21 compared BrECADD with escalated BEACOPP in patients aged 18–60 years (12–14). Nivolumab plus AVD (Nivo-AVD) is also supported by S1826 across age groups (15). In older adults, commonly defined as aged at least 60 years, physiological fitness and comorbidity are particularly relevant. Bleomycin toxicity and the tolerability of intensive chemotherapy limit extrapolation from younger cohorts. BV-AVD is an option, but the S1826 older-patient analysis favored Nivo-AVD for efficacy and tolerability in patients fit for anthracycline-based treatment (16). These advanced-stage trial results should not be generalized to frail patients unable to tolerate combination therapy.

Integrating ctDNA with PET-based response assessment in cHL is an active area of research, supported by published clinical and translational studies and discussed in previous reviews (8–10). Building on this literature, we synthesize the evidence for ctDNA as a biological classifier and a longitudinal measure of molecular response, and examine its potential contribution to combined assessment of stage, tumor biology, molecular residual disease, and PET response. The practical ctDNA readouts discussed in this review are summarized in **Table 1**.

**Table 1:** Practical ctDNA readouts in Hodgkin lymphoma.

Readout	Main question	Typical method	Current clinical role
Baseline genotype	What is the tumor?	Targeted NGS, CAPP-seq, PhasED-seq, WGS-based approaches	Biological classification, risk stratification, translational research
Tumor burden	How much molecular disease is present?	Quantitative NGS, fragmentomics, variant allele frequency or molecular burden estimates	Correlation with disease burden and TMTV; prognostic assessment
Early molecular response	How quickly does the tumor respond?	Serial ctDNA assessment after cycle 1 or cycle 2	Early response prediction; complements interim PET
MRD/molecular residual disease	What is left after treatment?	Tumor-informed or tumor-naive error-suppressed assays	Relapse-risk refinement; trial endpoint; not yet a stand-alone treatment trigger
Emerging resistance	What biology emerges under therapy?	Longitudinal NGS with clonal tracking	Mechanistic insight; potential future therapeutic targeting

CAPP-seq, cancer personalized profiling by deep sequencing; ctDNA, circulating tumor DNA; MRD, molecular residual disease (also termed minimal or measurable residual disease); NGS, next-generation sequencing; PET, positron emission tomography; PhasED-seq, phased variant enrichment and detection sequencing; TMTV, total metabolic tumor volume; WGS, whole-genome sequencing. The methods listed differ in validated breadth and sensitivity; the table does not imply equivalent performance or routine clinical endorsement.

## 2 Narrative review approach

This manuscript was developed as a narrative review for a clinically and translationally oriented readership. We aimed to synthesize methodological principles, landmark lymphoma studies, and cHL-specific evidence with published or publicly available molecular outcomes. It is not a systematic review or meta-analysis and does not claim compliance with PRISMA 2020, which provides reporting guidance for systematic reviews (17).

PubMed/MEDLINE and ClinicalTrials.gov were searched between 1 April and 30 June 2026, and the search was updated on 1 October 2026. Searches used the terms “Hodgkin lymphoma”, “ctDNA”, “cfDNA”, and “marker”, as well as the combination of “ctDNA” with “Hodgkin lymphoma”. Only English-language publications from 2010 through 2026 were eligible for inclusion. The authors performed the literature search and selected the publications. Priority was given to peer-reviewed studies, prospective biomarker analyses, large translational cohorts, landmark lymphoma ctDNA studies, and cHL-specific reports with interpretable molecular outcomes. Additional references were selected from the bibliographies of key articles when they informed methodology, standardization, or clinical interpretation.

The clinical evidence discussed comprises human studies with a defined lymphoma population, an interpretable plasma cfDNA/ctDNA assay, and findings relevant to tumor genotyping, molecular burden, response, residual disease, or prognosis. cHL studies form the main evidence base; FL, DLBCL, and myeloma studies provide disease-specific context. Methodological and mechanistic reports are used to explain assay performance and biology, rather than to establish treatment efficacy. The clinical synthesis does not use animal-only studies, isolated total cfDNA concentration measurements without evidence of tumor origin, or registry entries without molecular outcome data as proof of ctDNA clinical utility. Duplicate descriptions of a cohort are interpreted together to avoid treating them as independent patient populations.

Outcomes considered, where reported, include radiographic/metabolic response, ctDNA clearance or MRD status, relapse, progression-free survival (PFS), and overall survival (OS). Toxicity is considered when interpreting treatment-adaptation trials and the balance between disease control and treatment burden; it is not consistently reported as a ctDNA study endpoint. Study design, assay sensitivity, sampling time, treatment regimen, age range, and follow-up duration are considered when comparing findings. **Table 2** summarizes the principal cHL studies, with publication years and treatment context. Because this is a selective narrative synthesis, no pooled estimates or claim of exhaustive ascertainment are made.

**Table 2:** Selected Hodgkin lymphoma ctDNA studies, cohort-specific regimens, parent-trial protocols, and clinical implications.

A. Selected ctDNA studies and treatment context				
Study/framework (year)	Population and treatment regimen	Assay/approach	Key ctDNA finding	Clinical implication
ORIENT-1 (2020) (42)	Relapsed/refractory cHL; sintilimab monotherapy (anti-PD-1)	NGS-based ctDNA analysis	Early ctDNA decrease associated with radiographic response	Exploratory molecular response assessment during PD-1 blockade
Camus prospective monitoring (2021) (10)	Newly diagnosed cHL; ABVD/ABVD-like, escalated BEACOPP, or other regimens for older patients	Nine-gene targeted NGS; 0.5% VAF detection limit	Baseline variants in 70%; no detectable variants after cycle 2 with this assay	Feasibility established; low-level MRD assessment limited by sensitivity
Alig multicohort profiling (2024) (9)	366 patients from eight cohorts; Stanford contributed 33. Cohort-specific initial regimens are detailed in panel B; parent-trial schedules in panel C. Adult outcome analyses included previously untreated patients.	Plasma genomic profiling and PhasED-seq	H1/H2 genomic classes and prognostic molecular response associations	Biological classification and longitudinal risk refinement; no uniform treatment protocol
GHSG/Cologne classification (2024) (35)	Adult discovery cohort: 243 patients from HD21 and NIVAHL, as identified in the earlier report (46); EuroNet-PHL-C2 pediatric validation; HD21 outcome validation. Regimens: nivolumab/AVD in NIVAHL, BrECADD/eBEACOPP in HD21, and OEPA with risk- and response-adapted consolidation in EuroNet-PHL-C2 (panel C).	Genomic and microenvironmental classification	Three biologically defined classes; validation across adult and pediatric cohorts	Links genotype, viral associations, and microenvironment; treatment-specific validation needed
GHSG HD21 MRD-2 (2026) (43)	Advanced-stage cHL, aged 18-60; BrECADD or eBEACOPP: four cycles for PET2-negative and six for PET2-positive patients. PET-positive residual lesions received 30 Gy RT; MRD was assessed after cycle 2 (panel C).	Validated LymphoVista ctDNA sequencing assay; cycle-2 samples	MRD-2 adds prognostic information to PET-2 in a case-cohort analysis	Supports prospective testing of PET x MRD; not proof of benefit from ctDNA-guided therapy
FIL-RougeBIO/IOSI-EMA003 (registry; publication 2025) (44, 45)	FIL-RougeBIO: ABVD-based first-line therapy; IOSI-EMA003: treatment-naive cases and relapsed/refractory cases receiving checkpoint inhibitors	LyV4.0 CAPP-seq and genomic analyses	Two genetic instability patterns; whole-genome duplication associated with outcome during ABVD-based therapy	Published evidence beyond trial design; prospective ctDNA-triggered treatment benefit remains unproven
B. Initial treatment in the eight Alig cohorts (9).				
Cohort	n	Initial treatment reported in supplementary note table 3		
AHL2011	40	BEACOPP: 40		
Bellinzona	30	ABVD: 18; BEACOPP: 8; BrECADD: 4		
BREACH	102	ABVD: 35; BV-AVD: 67		
Leuven	29	ABVD: 23; other conventional chemotherapy: 6		
PAVD, Washington	30	Pembrolizumab + AVD: 30		
US pediatric trials	44	Pediatric regimens; AHOD1331 and cHOD17 are named in the Methods. Allocation between protocols and treatment arms was not established.		
PVAB	58	Prednisone, vinblastine, doxorubicin, and bendamustine: 58		
Stanford, California	33	ABVD: 10; BEACOPP: 1; BV-AVD: 6; BV + nivolumab: 4; BV + nivolumab + ipilimumab: 1; pembrolizumab + AVD: 3; Stanford V: 7; other regimen: 1		
Total	366	Eight cohorts; counts refer to initial treatment only.		
C. Treatment schedules, response assessment, and radiotherapy in the parent trials.				
Parent trial	Chemotherapy/number of cycles	PET/response assessment	Radiotherapy	
AHL2011 (38)	Six planned cycles. After two eBEACOPP cycles, the PET-adapted arm could switch to ABVD according to PET2; further decisions followed PET4.	PET after cycles 2 and 4; final response assessment.	No routine consolidative RT in this strategy.	
BREACH (47)	Four cycles of ABVD or BV-AVD.	PET2; PET4 for PET2-positive patients; final PET after RT.	30 Gy INRT in both arms, without a boost.	
PAVD/APVD (48)	Concurrent pembrolizumab + AVD; 2–6 cycles permitted. In the 30-patient parent trial: 2 cycles (n=5), 4 (n=3), and 6 (n=22).	PET after two cycles and after systemic treatment.	Preplanned consolidative RT was permitted and administered to 3/30 patients.	
PVAB (49)	Six planned cycles, every 21 days; two additional cycles after cycle 4 were given for complete or partial response.	CT after four cycles; final PET after six cycles.	RT was not allowed during protocol treatment.	
AHOD1331 (50)	Five 21-day cycles of BV-AVEPC versus ABVE-PC.	PET after two cycles; end-of-chemotherapy PET for slow-responding lesions, with further PET after RT when indicated.	21 Gy ISRT to slow-responding lesions and initial large mediastinal disease; a 9 Gy boost for incomplete metabolic response after cycle 5.	
cHOD17 (51)	Low risk: two BEABOVP cycles; intermediate risk: three BEABOVP cycles; high risk: two AEPA followed by four CAPDac cycles.	Response assessment after two cycles guides response-adapted treatment.	After chemotherapy, RT is limited to involved nodes without an adequate response after two cycles.	
NIVAHL (52)	Concurrent arm: four N-AVD cycles. Sequential arm: four nivolumab doses, then two N-AVD and two AVD cycles.	First PET after two N-AVD cycles or four nivolumab doses; reassessment after systemic treatment.	Planned 30 Gy ISRT in both arms.	
HD21 (12, 53)	BrECADD or eBEACOPP: four total cycles if PET2-negative, six if PET2-positive.	PET after two cycles. After chemotherapy, PET is mandatory for suspected active residual disease and optional otherwise.	30 Gy to PET-positive residual lesions after chemotherapy.	
EuroNet-PHL-C2: TL-1 (54)	Two OEPA cycles; one additional COPDAC-28 cycle for an adequate ERA response.	ERA/PET after two OEPA cycles.	Adequate ERA: omit RT. Inadequate ERA: 19.8 Gy RT.	
EuroNet-PHL-C2: TL-2 (54)	Two OEPA + two COPDAC-28 or DECOPDAC-21 cycles (four total).	ERA/PET after two OEPA cycles; LRA/PET after consolidation for inadequate ERA responders.	Adequate ERA: omit RT. Inadequate ERA, COPDAC-28: 19.8 Gy to initially involved sites + 10 Gy boost to LRA PET-positive lesions. DECOPDAC-21: 28.8 Gy only to LRA PET-positive sites; omit RT if LRA-negative.	
EuroNet-PHL-C2: TL-3 (54)	Two OEPA + four COPDAC-28 or DECOPDAC-21 cycles (six total).	ERA/PET after two OEPA cycles; LRA/PET after consolidation for inadequate ERA responders.	Same response- and arm-specific RT rules as TL-2.	

Source: Alig et al., Supplementary Note Table 3 (PDF pages 9-10; printed pages 8-9) (9). These are starting regimens; subsequent ABVD-to-AVD changes or escalation to BEACOPP are not included in the counts.

Interpretation: panel B reports observed initial regimens in the ctDNA cohort; panel C describes parent-trial treatment plans. The PAVD/APVD cycle counts and RT use are reported for its 30-patient parent trial. Protocol schedules do not establish the cycles or RT actually delivered to every participant in the ctDNA analyses. The AHOD1331/cHOD17 allocation of the 44 US pediatric patients, GHSG arm-specific numbers in the corrected supplement, and individual cycle counts and RT in the Stanford, Bellinzona, and Leuven center cohorts remain unverified. The Heger erratum documents replacement of an outdated data supplement (55).

(panels A–C): ABVD, doxorubicin, bleomycin, vinblastine, dacarbazine; ABVE-PC, doxorubicin, bleomycin, vincristine, etoposide, prednisone, cyclophosphamide; AEPA, brentuximab vedotin, etoposide, prednisone, doxorubicin; APVD/PAVD, pembrolizumab with AVD; AVD, doxorubicin, vinblastine, dacarbazine; BEABOVP, bendamustine, etoposide, doxorubicin, bleomycin, vincristine, vinblastine, prednisone; BEACOPP, bleomycin, etoposide, doxorubicin, cyclophosphamide, vincristine, procarbazine, prednisone; BrECADD, brentuximab vedotin, etoposide, cyclophosphamide, doxorubicin, dacarbazine, dexamethasone; BV, brentuximab vedotin; BV-AVD, BV with AVD; BV-AVEPC, BV, doxorubicin, vincristine, etoposide, prednisone, cyclophosphamide; CAPDac, cyclophosphamide, brentuximab vedotin, prednisone, dacarbazine; CAPP-seq, cancer personalized profiling by deep sequencing; cHL, classical Hodgkin lymphoma; COPDAC-28, cyclophosphamide, vincristine, prednisone, dacarbazine on a 28-day schedule; CT, computed tomography; ctDNA, circulating tumor DNA; DECOPDAC-21, doxorubicin, etoposide, cyclophosphamide, vincristine, prednisone, dacarbazine on a 21-day schedule; eBEACOPP, escalated BEACOPP; ERA, early response assessment; FIL, Fondazione Italiana Linfomi; GHSG, German Hodgkin Study Group; Gy, gray; INRT, involved-node radiotherapy; IOSI, Oncology Institute of Southern Switzerland; ISRT, involved-site radiotherapy; LRA, late response assessment; MRD-2, molecular residual disease after two treatment cycles; N-AVD, nivolumab with AVD; NGS, next-generation sequencing; OEPA, vincristine, etoposide, prednisone, doxorubicin; PD-1, programmed cell death protein 1; PET, positron emission tomography; PET2/PET4, PET after two/four treatment cycles; PhasED-seq, phased variant enrichment and detection sequencing; PVAB, prednisone, vinblastine, doxorubicin, bendamustine; RT, radiotherapy; TL, treatment level; VAF, variant allele fraction.

ClinicalTrials.gov entries were used only to describe trial design or ongoing biomarker studies. Registry information was not treated as evidence of clinical utility unless supported by published or publicly available ctDNA outcome data. Because the field is evolving rapidly, ongoing trials without interpretable ctDNA results are discussed only in the context of future directions. This distinction is essential: ctDNA-guided treatment decisions require not only analytical validity and prognostic association, but also prospective evidence that acting on the biomarker improves patient outcomes.

## 3 Why Hodgkin lymphoma is an ideal ctDNA disease

cHL has a distinctive biological architecture. Malignant HRS cells usually constitute only a small minority of the tumor mass, whereas the bulk of the lesion consists of reactive immune and stromal cells (9, 18, 19). This low malignant-cell fraction complicates bulk tissue sequencing and may limit detection of tumor-derived alterations unless malignant cells are enriched or sufficiently sensitive sequencing methods are used (9).

### 3.1 Tumor microenvironment and tumor-associated macrophages

The cHL microenvironment contains CD4-positive helper and regulatory T cells, CD8-positive cytotoxic T cells, B cells, plasma cells, macrophages, dendritic cells, eosinophils, neutrophils, mast cells, fibroblasts, and vascular/stromal elements. Their abundance and spatial organization vary between tumors. HRS-associated chemokines, including CCL17/TARC and CCL22, help recruit T cells, while cytokine signaling sustains reciprocal interactions between HRS cells and the inflammatory niche. Antitumor effector cells may therefore be present without producing effective tumor clearance (9, 11, 18).

Tumor-associated macrophages (TAMs) contribute to immune regulation and tissue remodeling. Increased CD68-positive macrophage content was associated with treatment failure and shorter survival in the landmark study by Steidl et al. (19). Spatial profiling subsequently showed that PD-L1-positive macrophages cluster around HRS cells and contact PD-1-positive T cells, supporting a local immune-evasion mechanism (18). TAM abundance and phenotype are thus potential prognostic markers and therapeutic targets. Their clinical use is limited by differences in markers, staining, spatial sampling, and thresholds; a CD68 or CD163 count is not a validated stand-alone treatment-selection test. PD-1 blockade can disrupt this immunosuppressive network, whereas direct macrophage depletion or reprogramming remains investigational in cHL (15, 18).

In contrast, plasma ctDNA can be abundant in cHL. Non-invasive genomic profiling has shown that plasma may capture HRS-derived alterations more effectively than bulk tissue sequencing in many patients (9, 10). Nonmalignant inflammatory cells contribute background cfDNA and can influence extracellular processing of tumor-derived DNA. Alig et al. linked characteristic cHL ctDNA fragmentation and enhanced shedding to DNASE1L3 expressed mainly by dendritic cells in the microenvironment, rather than by HRS cells themselves (9). Thus, a microenvironmental influence on ctDNA release should be distinguished from the cellular origin of tumor-specific mutations.

The clinical implication is important: in cHL, ctDNA is not simply a more convenient substitute for tissue analysis. It may provide information that tissue-based workflows struggle to obtain routinely, including representative tumor genotyping, molecular classification, and serial MRD assessment (9).

### 3.2 PET assessment and quantitative metabolic burden

FDG-PET/CT is the reference imaging method for staging and metabolic response assessment in FDG-avid cHL. The Deauville five-point scale compares lesion uptake with mediastinal blood pool and liver: score 1, no uptake above background; score 2, uptake no greater than mediastinum; score 3, uptake above mediastinum but no greater than liver; score 4, moderately above liver; and score 5, markedly above liver and/or new lesions attributable to lymphoma. Under Lugano response criteria, scores 1–3 usually indicate complete metabolic response, although individual de-escalation trials may use a stricter threshold. Scores 4–5 require comparison with baseline: reduced uptake can indicate partial metabolic response during treatment, whereas increasing uptake or lymphoma-related new lesions suggests progression (3).

Baseline quantitative PET measures provide additional burden estimates. SUVmax is the highest voxel uptake and describes focal intensity; TMTV is the sum of metabolically active tumor volumes across lesions; and total lesion glycolysis (TLG) sums lesion volume multiplied by lesion SUVmean. TMTV and TLG integrate disease extent, and baseline TMTV added prognostic information in the H10 cohort (20). A separate cohort evaluated the prognostic contribution of SUVmax, TMTV, and TLG (21). During therapy, changes in uptake, including delta SUVmax, and residual metabolic volume may further describe response. Their interpretation at interim and end-of-treatment assessments requires harmonized acquisition, uptake times, reconstruction, and lesion segmentation, with regimen-specific validation. No universally interchangeable quantitative threshold currently replaces Deauville-based response assessment. PET localizes disease and informs radiotherapy planning, while ctDNA measures tumor-derived molecular signal without identifying its anatomical site; their respective strengths explain the rationale for combined assessment (3, 10, 20, 21).

## 4 Pre-analytical and analytical requirements

The clinical interpretability of ctDNA depends on rigorous pre-analytical standardization. Blood collected into EDTA tubes should be processed rapidly, typically within 2–4 hours, to avoid leukocyte lysis and contamination with genomic DNA. When processing delays are expected, stabilizing tubes such as Streck Cell-Free DNA BCT or equivalent platforms are preferred. Plasma preparation should include double centrifugation to remove residual cells and debris, followed by aliquoting and storage at -80 °C until extraction (5, 22).

Assay selection should match the clinical question. Droplet digital PCR offers high sensitivity and absolute quantification for predefined variants, such as the recurrent XPO1 E571K mutation, when an informative target is available. Targeted NGS panels can identify variants in genes such as SOCS1, but the heterogeneity of SOCS1 mutations makes a single hotspot assay unsuitable for universal monitoring. CAPP-seq, PhasED-seq, duplex sequencing, and genome-wide approaches differ in breadth and sensitivity; not every platform provides equivalent detection of copy-number changes or structural variants. These methods can support genotyping, clonal tracking, or MRD assessment according to their validated capabilities (5, 7–10, 22).

For serial monitoring, consistency is critical. The same validated platform, similar sampling time points, and harmonized bioinformatic pipelines should be used whenever possible. Dynamic changes in ctDNA burden, rather than isolated single-time-point positivity, are often the most clinically meaningful readout. Error suppression and matched germline or cellular DNA controls are essential to distinguish tumor-derived alterations from clonal hematopoiesis and sequencing artifacts. Practical approaches include sequencing matched leukocyte or buffy coat DNA, filtering recurrent clonal hematopoiesis-associated variants, interpreting variant allele fractions in the context of tumor burden and fragment patterns, and predefining assay-specific limits of detection and positivity thresholds (5, 22).

The proposed laboratory workflow, including controls and reporting of assay sensitivity, is shown in **Figure 1**.

**Figure 1:**
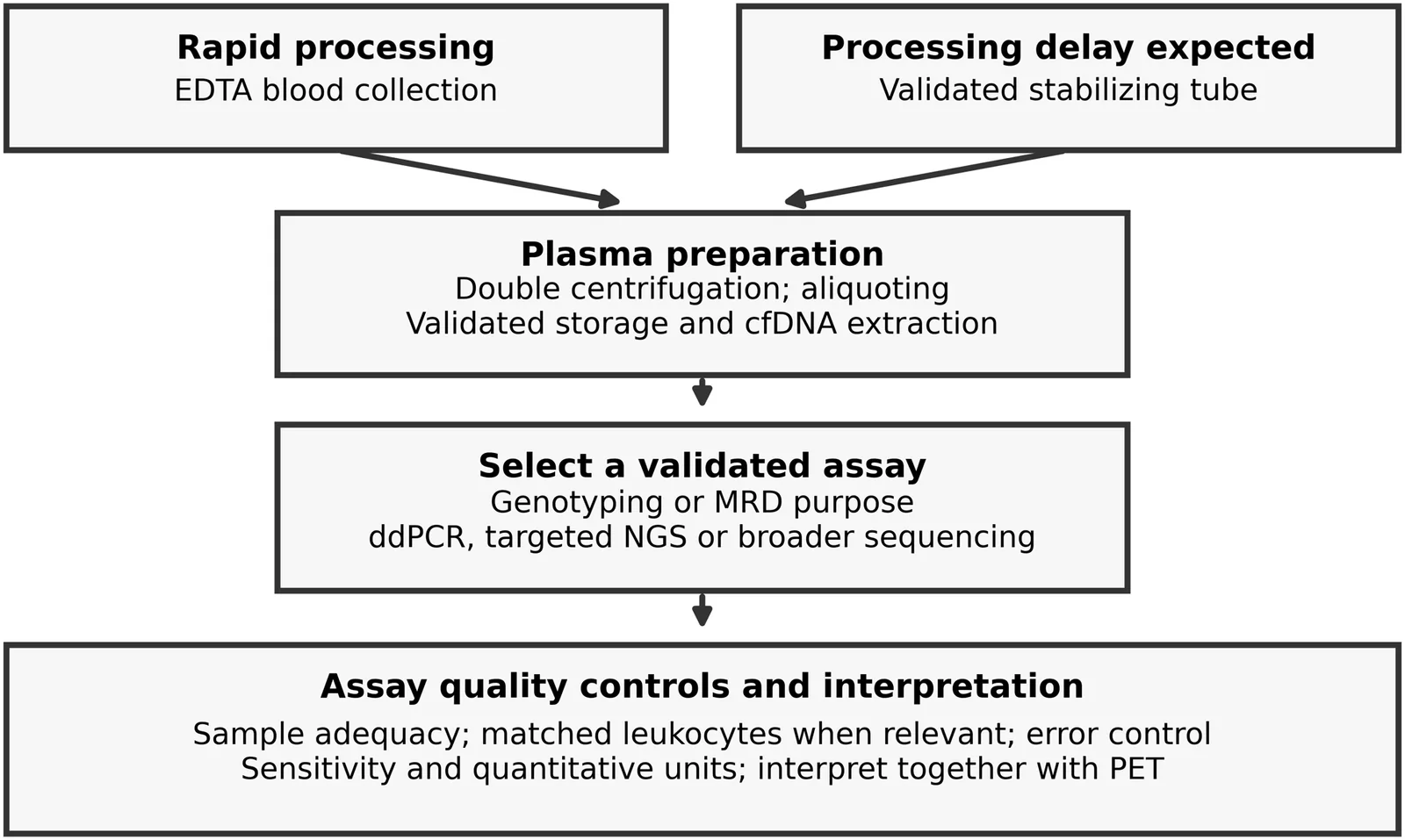
Clinical and laboratory workflow for ctDNA assessment in Hodgkin lymphoma. Peripheral blood is collected into EDTA tubes for rapid processing or validated stabilizing tubes when processing is delayed. Plasma is prepared by double centrifugation, aliquoted, and stored and extracted according to validated procedures. The assay is selected and analytically validated for the intended genotyping or MRD application. Interpretation considers sample adequacy, matched leukocyte controls where relevant, error control, sensitivity, quantitative units, and PET findings (5, 22). cfDNA, cell-free DNA; ctDNA, circulating tumor DNA; ddPCR, droplet digital polymerase chain reaction; EDTA, ethylenediaminetetraacetic acid; MRD, molecular residual disease; NGS, next-generation sequencing; PET, positron emission tomography.

## 5 ctDNA across hematologic malignancies: context for Hodgkin lymphoma

### 5.1 Follicular lymphoma and diffuse large B-cell lymphoma

Follicular lymphoma (FL) and diffuse large B-cell lymphoma (DLBCL) provide complementary clinical precedents for ctDNA assessment. In FL, circulating DNA measurements correlate with disease burden and TMTV, and molecular monitoring can identify early progression; however, an indolent course and heterogeneous shedding complicate intervention thresholds (23, 24). In DLBCL, baseline ctDNA and early molecular response after one or two treatment cycles predict outcome, while serial monitoring can detect relapse before conventional clinical assessment (25–27). These findings support adding a molecular measure of burden and response to imaging, but assay thresholds and treatment triggers cannot be transferred directly from either disease to cHL.

### 5.2 Multiple myeloma

In multiple myeloma, ctDNA can sample genomic heterogeneity beyond a single marrow site and support non-invasive assessment of mutations, copy-number alterations, and clonal evolution. Whole-exome sequencing has demonstrated substantial concordance between plasma and marrow tumor genomes (28). Its MRD role is less established: Mazzotti et al. found that plasma monitoring based only on immunoglobulin rearrangements did not correlate adequately with marrow MRD (29). ctDNA therefore remains an investigational complement to marrow-based next-generation flow/NGS and imaging, particularly for spatially heterogeneous disease, rather than a validated substitute for these assessments.

### 5.3 Leukemias, immunotherapy, and CAR-T-cell therapy

In leukemias, cfDNA-based ctDNA analysis is complicated by marrow involvement, circulating malignant cells, and clonal hematopoiesis. Nevertheless, cfDNA-based assays may complement bone marrow-based MRD in selected settings, particularly when repeated marrow sampling is difficult or extramedullary disease is suspected. In immunotherapy and CAR-T-cell therapy, ctDNA kinetics can help distinguish true progression from inflammatory or immune-related imaging changes and may detect relapse before clinical progression. Across these settings, the common principle is that ctDNA should be interpreted as an adjunct to established clinical and laboratory tools, not as an isolated decision-maker (30–34).

## 6 ctDNA in Hodgkin lymphoma: one assay, two clinical readouts

A central advantage of ctDNA in cHL is that one blood-based assay can generate two clinically relevant readouts. The first readout is genotypic and biological: it describes what the tumor is. This includes single nucleotide variants, copy-number alterations, structural patterns, viral associations, and molecular subtypes. The second readout is quantitative and dynamic: it describes what is left after treatment. This includes molecular disease burden, early molecular response, and MRD (8, 9, 35).

This dual-readout model is particularly well suited to cHL. At baseline, ctDNA may reveal biologically distinct disease classes that are not captured by histology or stage. During treatment, ctDNA kinetics may refine response assessment and identify discordance between imaging and molecular disease. Therefore, ctDNA should be understood as both a biological classifier and a response classifier.

**Figure 2** summarizes these complementary biological and longitudinal readouts. The breadth of information obtained depends on the selected assay.

**Figure 2:**
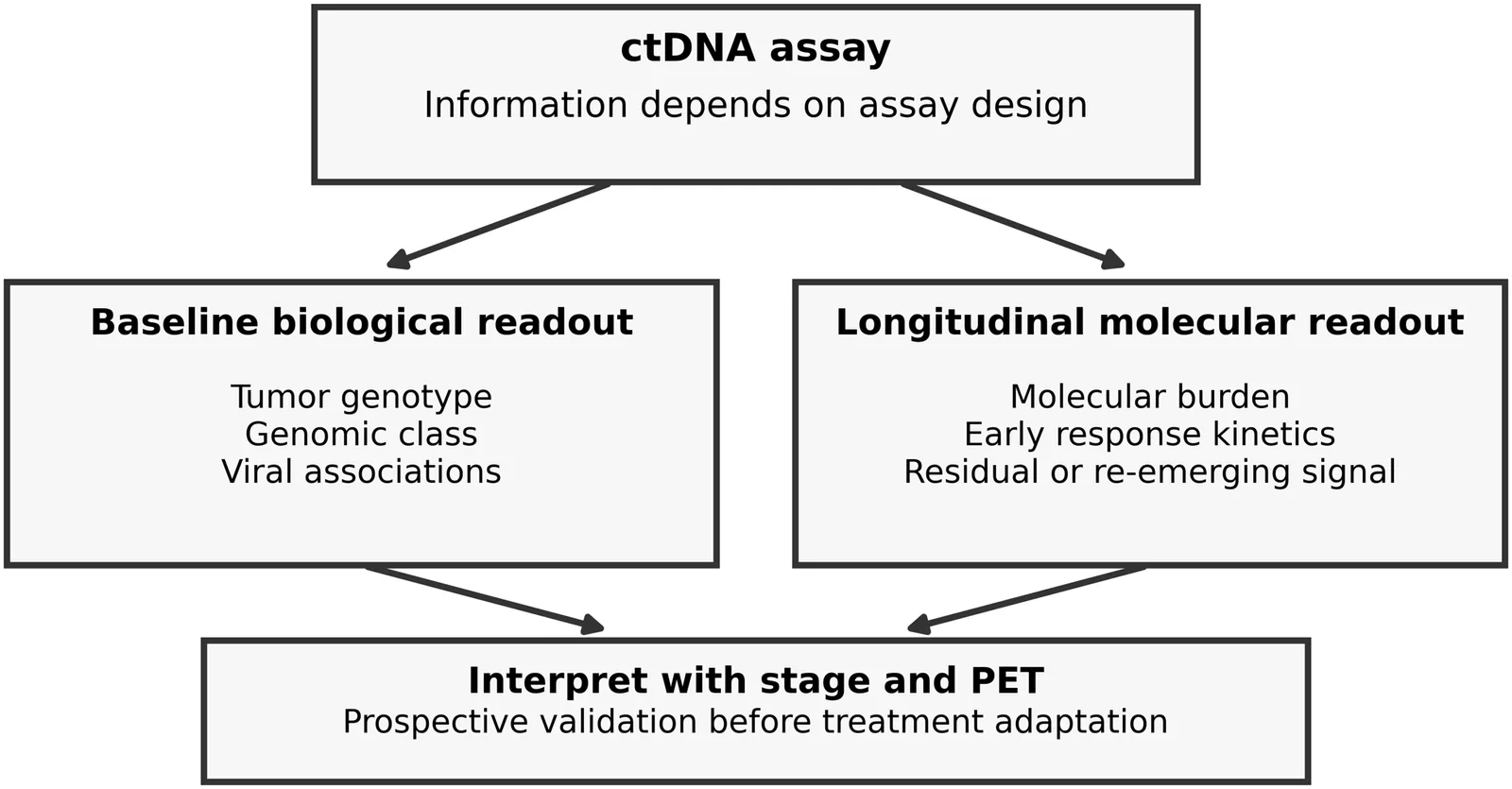
Biological and longitudinal ctDNA readouts in Hodgkin lymphoma. Depending on its design and validated performance, a ctDNA assay may provide a baseline biological readout of tumor genotype, genomic class, and viral associations, alongside serial molecular measurements of burden, early response, and residual or re-emerging disease. These complementary readouts are interpreted with stage and PET. Their use to direct treatment requires prospective validation (9, 35). ctDNA, circulating tumor DNA; PET, positron emission tomography.

## 7 Biological classification of Hodgkin lymphoma using ctDNA

### 7.1 Non-invasive genomic subtypes

Recent non-invasive profiling studies have shown that cHL can be divided into molecularly distinct subtypes using plasma ctDNA. The Stanford proposal identified H1 and H2 subtypes with different genomic patterns, viral associations, immune profiles, and clinical outcomes. Importantly, ctDNA profiling in this setting was not merely a surrogate for bulk tissue sequencing; it enabled molecular classification in a disease in which the malignant cell fraction in tissue is often too low for routine comprehensive genomic analysis (9).

These findings support the concept that the biological axis of cHL is readable non-invasively. This is a major shift: histology remains essential for diagnosis, but ctDNA can reveal biological heterogeneity within cHL that may eventually support risk-adapted and biology-adapted therapeutic strategies.

### 7.2 GHSG/Cologne biological classes and the tumor microenvironment

The GHSG/Cologne proposal further developed ctDNA-informed biological classification by integrating genomic features with the tumor microenvironment. Three biologically interpretable categories have been described: inflammatory immune escape HL, virally driven HL, and oncogene-driven HL. Inflammatory immune escape HL is characterized by immune escape-associated copy-number alterations and an inflammatory microenvironment. Virally driven HL is enriched for EBV and/or HHV6 associations and tends to show lower tumor mutational burden. Oncogene-driven HL is associated with recurrent alterations in oncogenic and immune signaling pathways (35).

This framework is clinically relevant because it links tumor genotype, viral oncogenesis, immune contexture, and prognosis. It also illustrates why ctDNA should be interpreted together with tissue-based or blood-derived information on the tumor microenvironment. In cHL, cfDNA and ctDNA reflect not only malignant HRS-cell biology, but also the biology of the inflammatory niche in which HRS cells survive. Immune escape, viral association, antigen-presentation defects, copy-number alterations, and the composition of the microenvironment are therefore interdependent dimensions of the same disease process rather than separate biomarkers.

### 7.3 Convergence, but not interchangeability, of current classification frameworks

Across independent groups, the emerging picture is one of convergence. Although different analytical methods and labels are used, studies increasingly suggest a reproducible biological structure in cHL: one axis related to mutational load and oncogene-driven biology, another related to chromosomal instability and copy-number alterations, with viral oncogenesis intersecting these categories. This convergence is encouraging, but it should not yet be equated with clinical actionability (9, 35).

These frameworks should not be considered interchangeable. Rather, they represent convergent but methodologically distinct attempts to define non-invasive biological structure in cHL. At present, biological classification using ctDNA is best considered research-ready and trial-ready. It can improve patient stratification, enrich translational analyses, and generate hypotheses for targeted therapy, but treatment selection based solely on baseline biological class remains investigational. The readiness of the main ctDNA applications for clinical use is summarized in **Table 3**.

**Table 3:** Readiness of ctDNA applications in Hodgkin lymphoma.

Application	Evidence strength	Current status	Key requirement before routine use
Baseline molecular classification	Strong translational evidence	Research-ready/trial-ready	Prospective validation of clinical actionability
Tumor burden estimation	Moderate to strong	Complementary to imaging	Standardized quantitative thresholds
Early molecular response	Moderate to strong	Promising trial biomarker	Harmonized sampling after cycle 1 or cycle 2
MRD after therapy	Strong prognostic rationale	Investigational for treatment decisions	Interventional trials testing ctDNA-guided action
PET x MRD integration	Emerging and clinically compelling	High-priority research direction	Prospective validation of discordant PET/MRD categories
Therapy selection by biological class	Preliminary	Not ready for routine use	Predictive biomarkers and targeted therapeutic options

ctDNA, circulating tumor DNA; MRD, molecular residual disease; PET, positron emission tomography. Readiness refers to the available evidence and does not constitute an endorsement of ctDNA-directed treatment outside a trial.

## 8 ctDNA-MRD as a dynamic response classifier

Response-adapted therapy is already central to cHL management. Interim FDG-PET, typically assessed after two cycles of treatment, remains the dominant dynamic classifier and has shaped de-escalation and escalation strategies in major clinical trials. However, PET is not a purely tumor-specific biomarker. Inflammation, infection, immune activation, and post-treatment tissue remodeling can generate false-positive or equivocal findings. Conversely, molecular residual disease may persist despite apparent metabolic response (3, 12, 15, 36).

The PET-adapted evidence base illustrates both the value and the limits of early metabolic response. In RATHL, patients with a negative PET after two ABVD cycles were randomized to continue ABVD or omit bleomycin; AVD reduced pulmonary toxicity with very similar PFS, although the initial analysis narrowly missed the prespecified non-inferiority boundary (14). In GHSG HD18, a negative PET after two escalated BEACOPP cycles supported reduction to four total cycles rather than six or eight, with less severe toxicity (37). AHL2011 similarly supported switching PET-2-negative patients from escalated BEACOPP to ABVD, maintaining disease control while reducing treatment burden (38).

Early-stage trials require a more cautious interpretation of de-escalation. EORTC/LYSA/FIL H10 did not establish non-inferiority of radiotherapy omission in PET-2-negative patients; in PET-2-positive patients, switching from ABVD to escalated BEACOPP plus radiotherapy improved PFS in the primary analysis (39). RAPID, using PET after three ABVD cycles, also failed to demonstrate non-inferiority of omitting radiotherapy despite favorable outcomes in both arms (40). In advanced-stage HD0801, PET-2 positivity triggered early salvage chemotherapy and autologous transplantation in a nonrandomized phase II strategy, demonstrating feasibility rather than definitive superiority over alternative escalation approaches (41).

These trials used different initial regimens, PET time points, and positivity thresholds. A ctDNA-guided strategy must therefore be evaluated within a defined treatment context; molecular negativity alone cannot justify importing a de-escalation rule from another trial (14, 37–41).

ctDNA-MRD addresses this gap by measuring tumor-derived molecular signal during and after therapy. In cHL, early reductions in ctDNA have been associated with treatment response, and persistent or re-emerging ctDNA has been associated with inferior outcomes. The GHSG HD21 MRD-2 analyses suggest that ctDNA after two cycles of BrECADD/eBEACOPP adds prognostic information even in the context of modern, highly effective regimens (10, 42, 43).

This does not mean that ctDNA-MRD should be considered a fifth, independent axis of classification. Rather, it upgrades the dynamic-response axis. PET describes metabolic response; ctDNA-MRD describes molecular response. Their integration may identify more precise risk groups than either modality alone.

## 9 Integrating PET and MRD: sharper risk stratification

The clinically most useful model is not PET versus ctDNA, but PET x MRD. Four response categories can be conceptualized. Patients who are PET-negative and MRD-negative are expected to represent a very low-risk group. Patients who are PET-positive and MRD-positive represent a high-risk group with concordant imaging and molecular evidence of residual disease. Patients who are PET-positive but MRD-negative form an important discordant group in which residual metabolic activity may reflect inflammation, sampling timing, low-volume disease below the assay threshold, or other biological and technical factors. Patients who are PET-negative but MRD-positive are also highly relevant, as they may harbor molecular residual disease that is radiographically occult (10, 43).

This framework is attractive for future trials because it can identify patients for whom escalation, de-escalation, biopsy, repeat imaging, or intensified molecular follow-up may be appropriate. However, discordant PET/MRD categories must not be overinterpreted outside prospective validation. The key clinical question is not whether ctDNA predicts outcome, but whether changing therapy on the basis of ctDNA improves the benefit-harm balance.

The conceptual PET x MRD framework is illustrated in **Figure 3**. A result should be considered MRD-negative only when the sample and assay are adequate and informative; an unevaluable specimen should not be assigned to a negative category.

**Figure 3:**
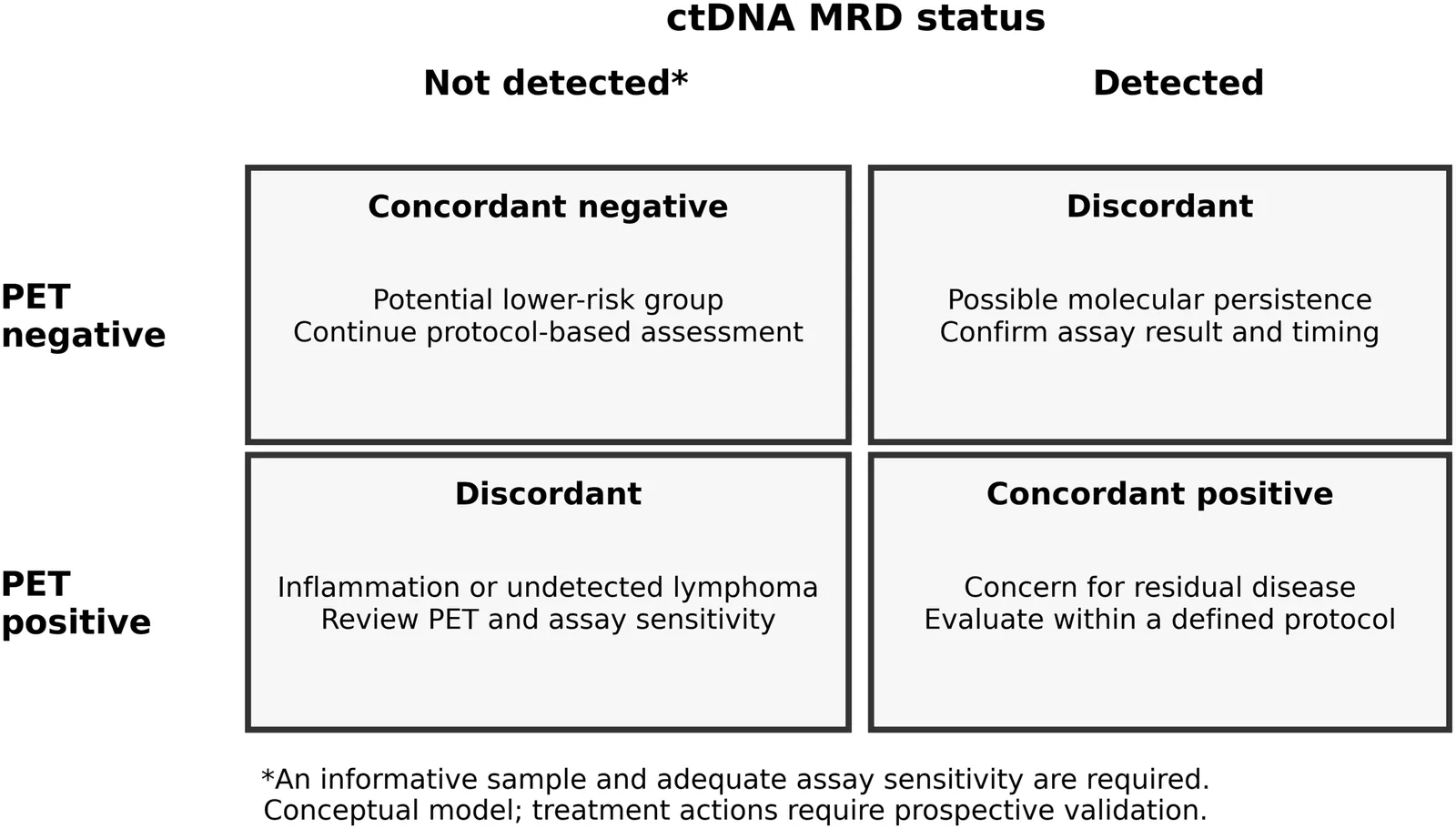
Conceptual integration of PET and ctDNA MRD. The four PET/ctDNA categories represent a hypothesis for prospective risk stratification. PET status must be defined for the assessment time point and treatment protocol. Concordant negative results may identify lower risk, whereas concordant positive results raise concern for residual disease. Discordant findings require clinical correlation, review of sampling and assay sensitivity, and, when indicated, repeat assessment or biopsy. A negative molecular result requires an informative sample and adequate assay sensitivity; unevaluable samples are excluded from the negative category. Undetectable ctDNA does not prove the absence of viable lymphoma. Treatment adaptation based on these categories remains investigational (3, 9, 43). ctDNA, circulating tumor DNA; MRD, molecular residual disease; PET, positron emission tomography.

## 10 Evidence from Hodgkin lymphoma studies

Several cHL studies demonstrate analytical feasibility and prognostic associations, although these do not by themselves establish clinical utility. ORIENT-1 evaluated ctDNA during sintilimab treatment in relapsed/refractory cHL; an early decrease was associated with radiographic response (42). Camus et al. prospectively assessed a nine-gene panel, including XPO1, in patients receiving ABVD/ABVD-like regimens, escalated BEACOPP, or other regimens for older patients. Baseline mutations were detected in 70% of patients. No variants remained detectable after two cycles with that assay, whose 0.5% VAF detection limit restricted assessment of low-level residual disease (10).

FIL-RougeBIO is a prospective observational substudy of patients receiving ABVD-based first-line therapy (44). Published genomic outcome data are now available: Pirosa et al. analyzed 297 patients from IOSI-EMA003 and FIL-RougeBIO, identifying two genetic instability patterns and an association between whole-genome duplication and adverse outcomes during ABVD-based treatment. The study also explored ctDNA in the interpretation of ambiguous PET/CT-positive lesions (45). These findings update the evidence beyond the registry description, but do not establish that changing therapy according to ctDNA improves outcomes.

Newer studies have expanded the field beyond targeted single-gene monitoring. Non-invasive genomic profiling has defined cHL subtypes with biological and prognostic relevance, while the HD21 MRD-2 analysis indicates that ctDNA complements PET-2 after BrECADD or escalated BEACOPP. The latter used an event-enriched case-cohort design, requiring appropriate weighting when extrapolating risk estimates to the trial population. These studies support ctDNA as a translational biomarker, but no cHL treatment algorithm should currently be changed solely on the basis of ctDNA outside a clinical trial (9, 35, 43, 45).

### 10.1 Age-related biology and limits of subgroup evidence

Age-specific biological analyses are available, but age-specific validation of ctDNA-guided treatment remains limited. In Alig et al., H1 tumors predominated in younger patients, whereas H2 tumors showed a bimodal age distribution with a second peak above 60 years and enrichment for EBV-positive/mixed-cellularity disease. An external gene-expression analysis also compared patients aged at least 65 years with younger patients (9). Heger et al. validated biological subtypes in 96 children and adolescents from EuroNet-PHL-C2 (35). These analyses address biological differences rather than proving equivalent MRD performance across ages.

Older adults were not represented beyond age 60 in the randomized HD21 population, limiting direct extrapolation of its MRD-2 findings to this group (12, 43). Camus et al. included patients up to age 86, but this small feasibility cohort does not establish a validated older-patient ctDNA threshold (10). The S1826 analysis in patients aged at least 60 years provides treatment outcome and safety data, rather than an age-specific ctDNA validation (16). Future studies should report age-stratified assay informativeness, clearance, PET/ctDNA concordance, and associations with PFS and OS, accounting for EBV status, regimen, comorbidity, treatment-related mortality, and clonal hematopoiesis.

## 11 What is ready for practice and what remains investigational?

ctDNA is suitable for translational research and exploratory clinical trial stratification. Potential applications include non-invasive tumor genotyping, molecular burden estimation, serial response monitoring, and investigation of discordant imaging findings. These uses should be distinguished from routine staging or response categorization, for which clinical assessment and Lugano-based FDG-PET/CT remain the standard. Neither ctDNA-guided treatment escalation nor de-escalation is supported by sufficient prospective interventional evidence in cHL (3, 8–10, 35, 42, 43, 45).

A biomarker that changes therapy must satisfy a higher evidentiary standard than a biomarker that predicts prognosis. It must be analytically reproducible, accessible and serially measurable, predictive of differential treatment benefit, and net beneficial when acted upon. This distinction is crucial in cHL, where cure rates are high and unnecessary escalation may increase acute and late toxicity, while inappropriate de-escalation may compromise disease control.

For staging, the main limitation is that plasma ctDNA cannot localize nodal or extranodal disease, distinguish anatomical stages, or replace histological confirmation. ctDNA burden is associated with stage and TMTV but is also affected by shedding, tissue turnover, and microenvironmental DNA processing. Low-volume or poorly shedding disease may yield an undetectable result, so molecular burden cannot currently substitute for Lugano/Ann Arbor staging (3, 9, 10).

For response assessment, assays differ in panel coverage, DNA input, error suppression, and limits of detection. A specimen without an informative baseline signal may not support longitudinal mutation tracking. A negative MRD test can reflect insufficient molecules or limited sensitivity, while clonal hematopoiesis or sequencing artifacts can produce false-positive calls. Timing relative to chemotherapy, immunotherapy, and PET also affects interpretation. There is no universally validated cHL definition of molecular response, positivity threshold, or rule for handling discordant PET/ctDNA findings across all regimens and age groups (5, 9, 10, 22, 43).

Implementation is further constrained by access to validated assays, cost and reimbursement, laboratory capacity, and turnaround times compatible with treatment decisions. Most published cHL evidence establishes feasibility or prognosis rather than benefit from a ctDNA-triggered intervention. These technical, biological, clinical, and operational barriers explain why routine ctDNA-based staging and response classification have not yet been adopted (5, 22, 43, 56).

## 12 Future directions

A practical route to implementation is a multicenter program combining analytical harmonization, external clinical validation, and prospective testing of biomarker-guided decisions. Baseline stage, PET response, ctDNA-MRD, genomic class, and microenvironmental features should be assessed together, with treatment changes evaluated in trials before adoption into routine care (**Figure 4**) (5, 9, 22, 35, 43).

**Figure 4:**
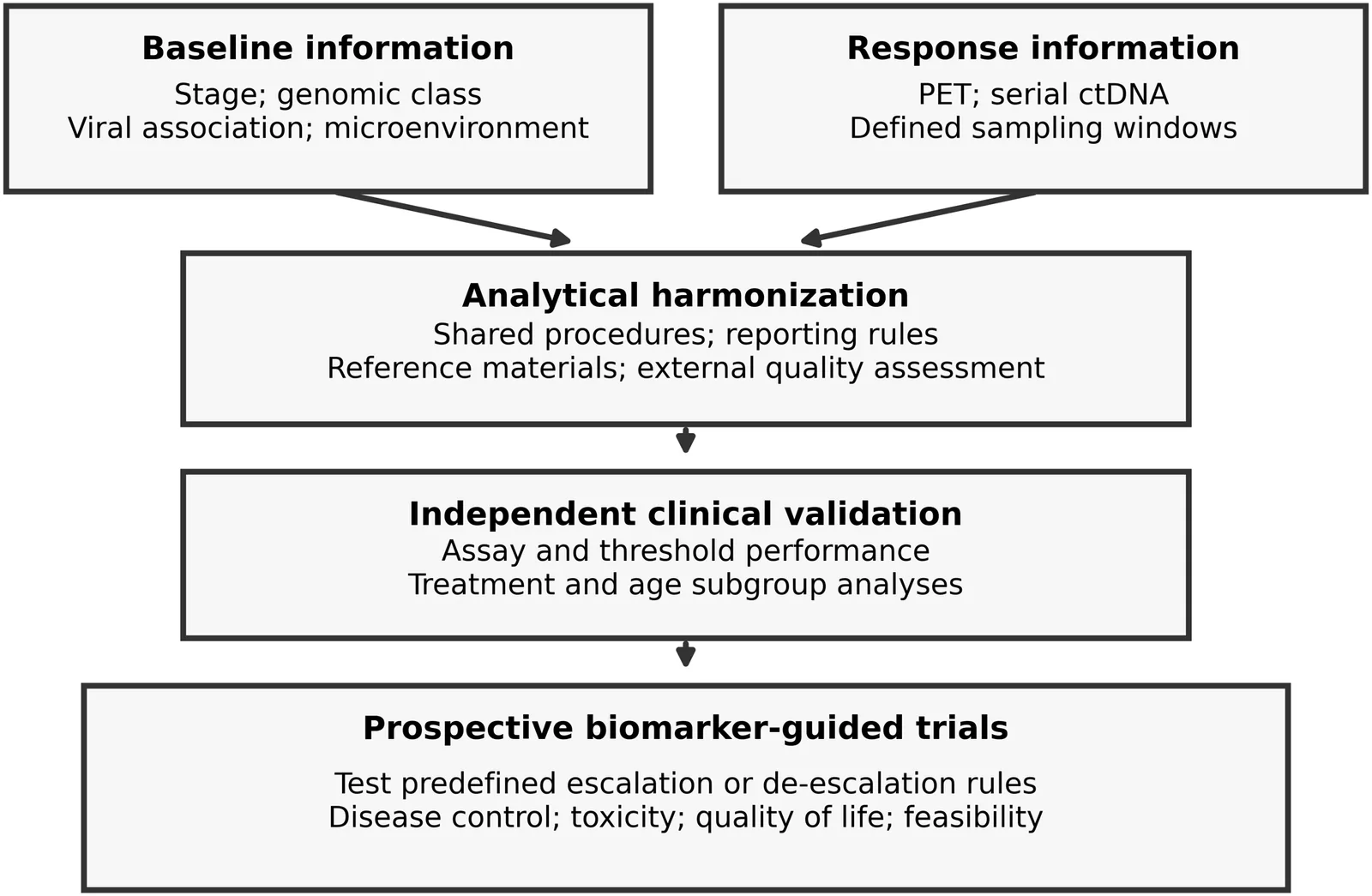
Proposed validation pathway for an integrated classifier in Hodgkin lymphoma. Baseline stage, genomic class, viral associations, and tumor microenvironment are combined with PET and serial ctDNA response measures. Analytical harmonization includes shared procedures, reporting rules, reference materials, and external quality assessment. Independent clinical validation evaluates assay and threshold performance across treatment and age groups. Prospective trials then test predefined biomarker-guided treatment rules and assess disease control, toxicity, quality of life, and feasibility. This is a proposed development pathway (5, 9, 22, 43). ctDNA, circulating tumor DNA; PET, positron emission tomography.

First, laboratories should adopt shared standard operating procedures for collection tubes, processing intervals, double centrifugation, plasma volume, storage, extraction, and freeze-thaw exposure. Assays should be validated separately for baseline genotyping and MRD, using reference materials and dilution series to establish sensitivity, specificity, precision, limits of detection and quantification, and performance at low DNA input. Matched leukocyte controls and error-suppression methods should be incorporated where appropriate. Blinded sample exchanges, external quality assessment, and cross-platform comparisons would test reproducibility rather than assuming that different assays are interchangeable (5, 9, 22, 43).

Second, clinical validation should use predefined sampling windows, for example before treatment, during an early cycle, after two cycles, and at the end of treatment, with blood collection aligned to the relevant PET examination. These are proposed trial time points, not a validated universal schedule. Reports should state sample adequacy, baseline informativeness, tracked variants, DNA input, plasma volume, quantitative units, assay-specific sensitivity, and whether ctDNA is detected, not detected, or not evaluable. Independent cohorts should validate thresholds within treatment and age strata, including adults aged 60 years or older. The recent lymphoma technology reviews reinforce the need to match each assay to its intended use (56–58).

Third, biomarker-stratified interventional trials should prespecify how each PET/ctDNA category is managed and compare that strategy with standard PET- and stage-adapted care. Possible trial actions include confirmatory sampling or biopsy for discordant findings, escalation for persistent molecular disease, and de-escalation for concordant metabolic and molecular remission. Primary clinical endpoints should assess disease control and toxicity; PFS, OS, late effects, quality of life, feasibility, and cost-effectiveness should also be measured. Randomization where feasible is needed to establish whether acting on ctDNA improves the benefit-harm balance, particularly in a highly curable disease.

## 13 Conclusions

ctDNA has moved cHL into a new molecular era. It enables non-invasive biological classification in a disease where tissue-based genomic profiling is intrinsically difficult, and it provides a serial molecular readout of response and MRD. This review synthesizes current evidence on the complementary roles of ctDNA and PET, relating baseline biological classification to serial molecular and metabolic response assessment.

The most mature near-term use of ctDNA in cHL is as an investigational molecular complement to PET-based risk stratification. Stage describes the anatomical distribution of disease, PET measures metabolic activity, and ctDNA characterizes tumor-derived molecular features and their persistence during or after therapy. Each modality has limitations, so combined assessment requires validated assays and prospective evaluation.

For now, ctDNA should strengthen clinical trials and translational studies. Routine staging, response categorization, or treatment adaptation based on ctDNA will require reproducible laboratory procedures, externally validated thresholds, and prospective evidence that acting on the result improves patient outcomes.
